# The effect of nanomaterials on embryonic stem cell neural differentiation: a systematic review

**DOI:** 10.1186/s40001-023-01546-0

**Published:** 2023-12-09

**Authors:** Ramyar Rahimi Darehbagh, Mozaffar Mahmoodi, Nader Amini, Media Babahajiani, Azra Allavaisie, Yousef Moradi

**Affiliations:** 1 Student Research Committee, Kurdistan University of Medical Sciences, Sanandaj, Iran; 2 Nanoclub Elites Association, Tehran, Iran; 3 Cellular and Molecular Research Center, Research Institute for Health Development, Kurdistan University of Medical Sciences, Sanandaj, Iran; 4 Department of Molecular Medicine, Faculty of Medicine, Kurdistan University of Medical Sciences, Sanandaj, Iran; 5 Environmental Health Research Center, Research Institute for Health Development, Kurdistan University of Medical Sciences, Sanandaj, Iran; 6 Department of Epidemiology and Biostatistics, Faculty of Medicine, Kurdistan University of Medical Sciences, Sanandaj, Iran; 7 Department of Anatomical Sciences, School of Medicine, Sanandaj, Iran

**Keywords:** Nanomaterials, Embryonic stem cell, Neural differentiation

## Abstract

**Background:**

Humans’ nervous system has a limited ability to repair nerve cells, which poses substantial challenges in treating injuries and diseases. Stem cells are identified by the potential to renew their selves and develop into several cell types, making them ideal candidates for cell replacement in injured neurons. Neuronal differentiation of embryonic stem cells in modern medicine is significant. Nanomaterials have distinct advantages in directing stem cell function and tissue regeneration in this field. We attempted in this systematic review to collect data, analyze them, and report results on the effect of nanomaterials on neuronal differentiation of embryonic stem cells.

**Methods:**

International databases such as PubMed, Scopus, ISI Web of Science, and EMBASE were searched for available articles on the effect of nanomaterials on neuronal differentiation of embryonic stem cells (up to OCTOBER 2023). After that, screening (by title, abstract, and full text), selection, and data extraction were performed. Also, quality assessment was conducted based on the STROBE checklist.

**Results:**

In total, 1507 articles were identified and assessed, and then only 29 articles were found eligible to be included. Nine studies used 0D nanomaterials, ten used 1D nanomaterials, two reported 2D nanomaterials, and eight demonstrated the application of 3D nanomaterials. The main biomaterial in studies was polymer-based composites. Three studies reported the negative effect of nanomaterials on neural differentiation.

**Conclusion:**

Neural differentiation is crucial in neurological regenerative medicine. Nanomaterials with different characteristics, particularly those cellular regulating activities and stem cell fate, have much potential in neural tissue engineering. These findings indicate a new understanding of potential applications of physicochemical cues in nerve tissue engineering.

**Graphical Abstract:**

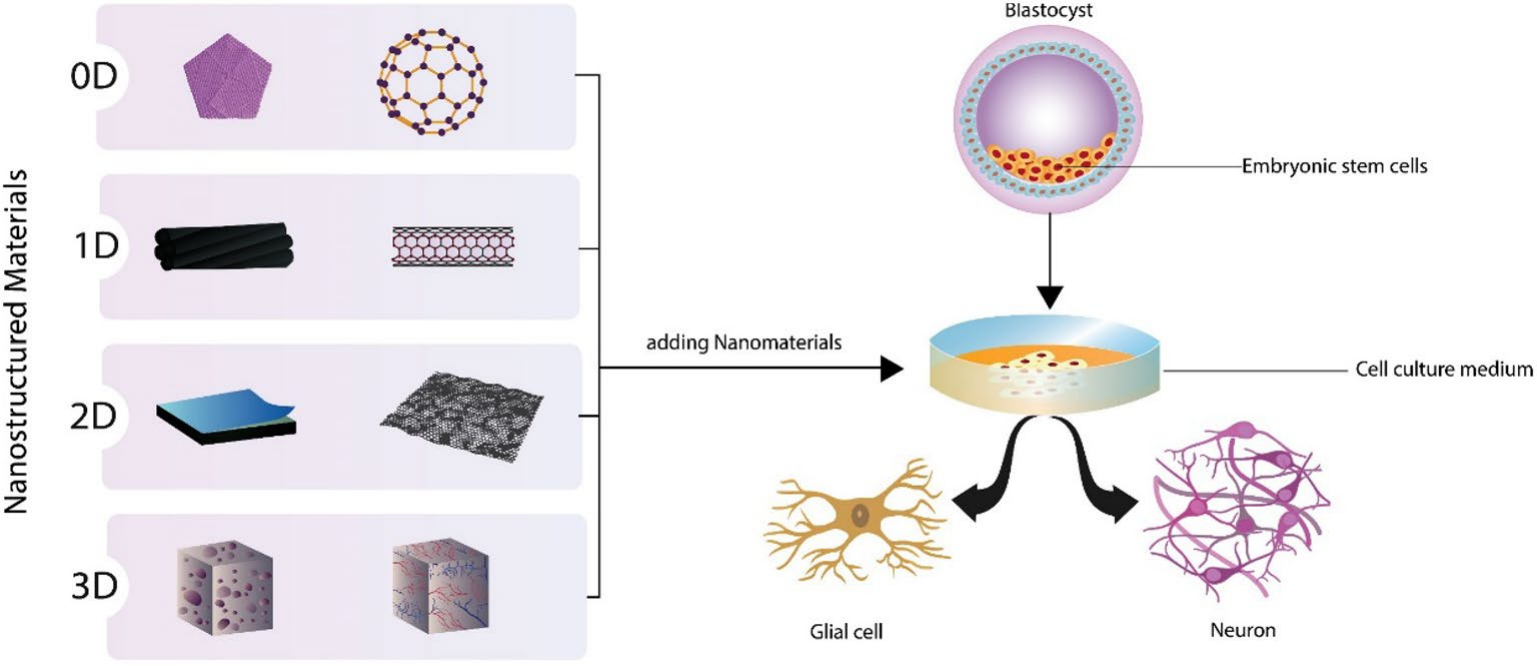

## Introduction

Neurons and glial cells are the two basic types of cells in the nervous system. Neurons are distinguished from other cells by several characteristics, the most important of which is communicating with other cells through synapses. Hundreds of different types of neurons exist in the nervous system of a single species, such as humans, with a vast range of morphologies and functions. Sensory neurons translate physical stimuli such as light and sound into neural impulses, whereas motor neurons translate neural signals into muscle or gland action. Most neurons, however, develop centralized structures (the brain and ganglia) in many animals, receiving all of their input from other neurons and giving all of their output to other neurons [1, 2]. Glial cells are non-neuronal cells in the nervous system, which provide support and nutrition, regulate homeostasis, produce myelin, and aid signal transmission. Glial cells perform various essential functions, such as supporting and holding neurons in place, supplying nutrients to neurons, electrically insulating neurons, destroying infections and removing dead neurons, and providing guiding cues to direct neuron axons to their destinations. Glial cells (oligodendrocytes of the central nervous system and Schwann cells of the peripheral nervous system) produce layers of myelin. This fatty substance wraps around axons and provides electrical insulation, allowing them to transfer action potentials much faster and more efficiently. Microglia and astrocytes, two types of glial cells, have recently been discovered to play an essential role as resident immune cells in the central nervous system [3–5]. The repair or replacement of nerve cells destroyed by injuries or diseases is required for nervous system regeneration. While lower organisms have a large capacity for neural regeneration, evolutionarily higher organisms, such as humans, have a limited ability to repair nerve cells, which poses substantial challenges in treating nervous system injuries and diseases. Regardless of the underlying cause of nervous system injuries, the result is often an inability of nerve cells to transmit neural impulses to specific nervous system sections. One of the three types of nervous system repair is required to regain functionality. Damaged neuronal axons can regenerate, whereas the rest of the neuron, including the cell body, is unaffected. Other approaches include repairing damaged nerve cells and creating new neurons to replace lost ones. While these three processes of nervous system repair potentially might repair all types of damage and degeneration, they are often successful in only selected parts of the nervous system [6, 7].

**Fig. 1:**
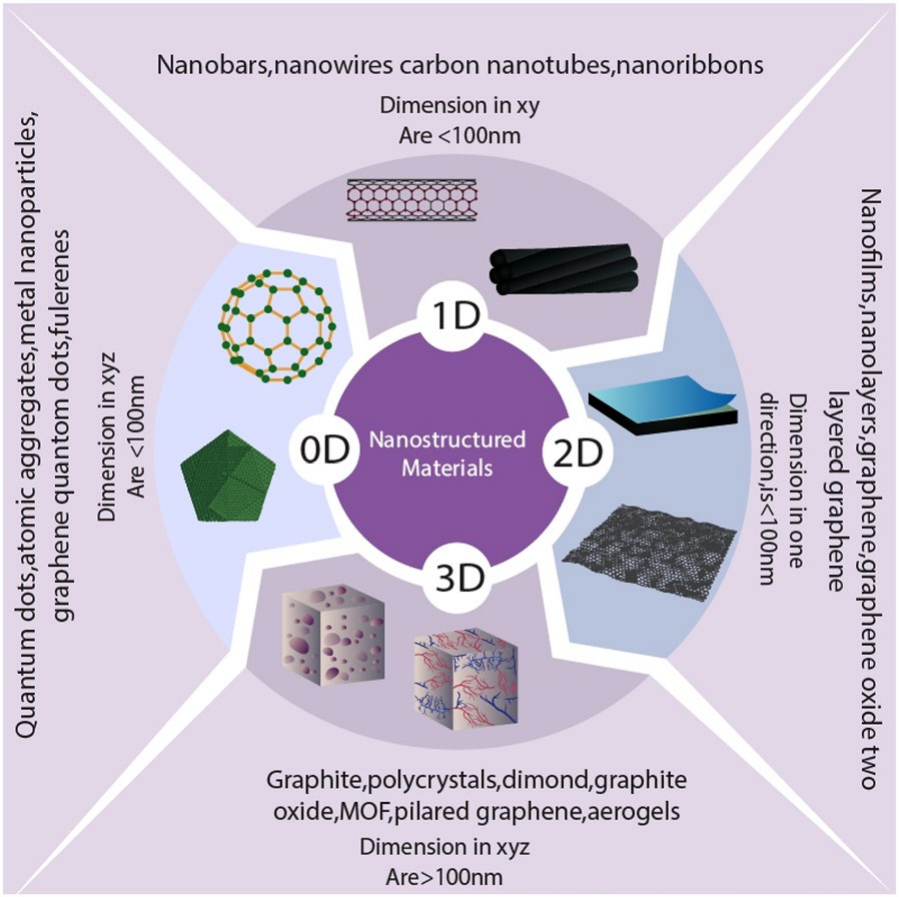
The dimensional nanostructure classification

Stem cells are good tools for neural repair. They are identified by the potential to renew their selves and develop into several cell types, making them ideal candidates for cell replacement in injured neurons. Adult stem cells from the hippocampus and subventricular zone have traditionally been used as a source of neural stem cells for replacement. New and intriguing ways for neural cell replacement are being developed because of the advancement of pluripotent stem cells, such as human embryonic stem cells (hESCs) and human induced pluripotent stem cells (iPSCs) [6]. Embryonic stem cells (ESCs) generated from the inner cell mass of a blastocyt have pluripotency, allowing them to reproduce forever and develop into all three embryonic germ layer derivatives [8]. The differentiation of ESCs into other somatic cell types, particularly neural progenitor cells (NPCs), has been used as an in vitro model to research neurogenesis in early human development, including the molecular mechanisms of proliferation and differentiation. The plasticity and self-renewal capabilities of ESCs paved the path for stem cell transplantation, regenerative medicine, and tissue engineering [9, 10]. The control and management of differentiation of cells into particular cell types is essential for the clinical application of stem cells, particularly in cell therapy and tissue engineering. However, the progress of stem cell differentiation for stem cell treatment is constrained by the poor differentiation efficiency and success rate. It is crucial to allow committed differentiation of ESCs into specific lineages before implantation for safe use in cell-based therapies because undifferentiated ESCs in vivo increases the teratoma risk. Therefore, techniques to increase the effectiveness of directed differentiation of stem cells into particular cell types must be immediately developed [11]. Growth factors, hormones, minor chemicals, and extracellular matrix are examples of biological cues and biomaterials which might influence the stem cell fate of differentiation and pluripotency. One of these biomaterials is nanomaterials, which, due to their small size, simplicity in synthesis, and flexibility in surface functionalization, have been widely used to control the behavior of cells [12] (Fig. 1).

**Fig. 2:**
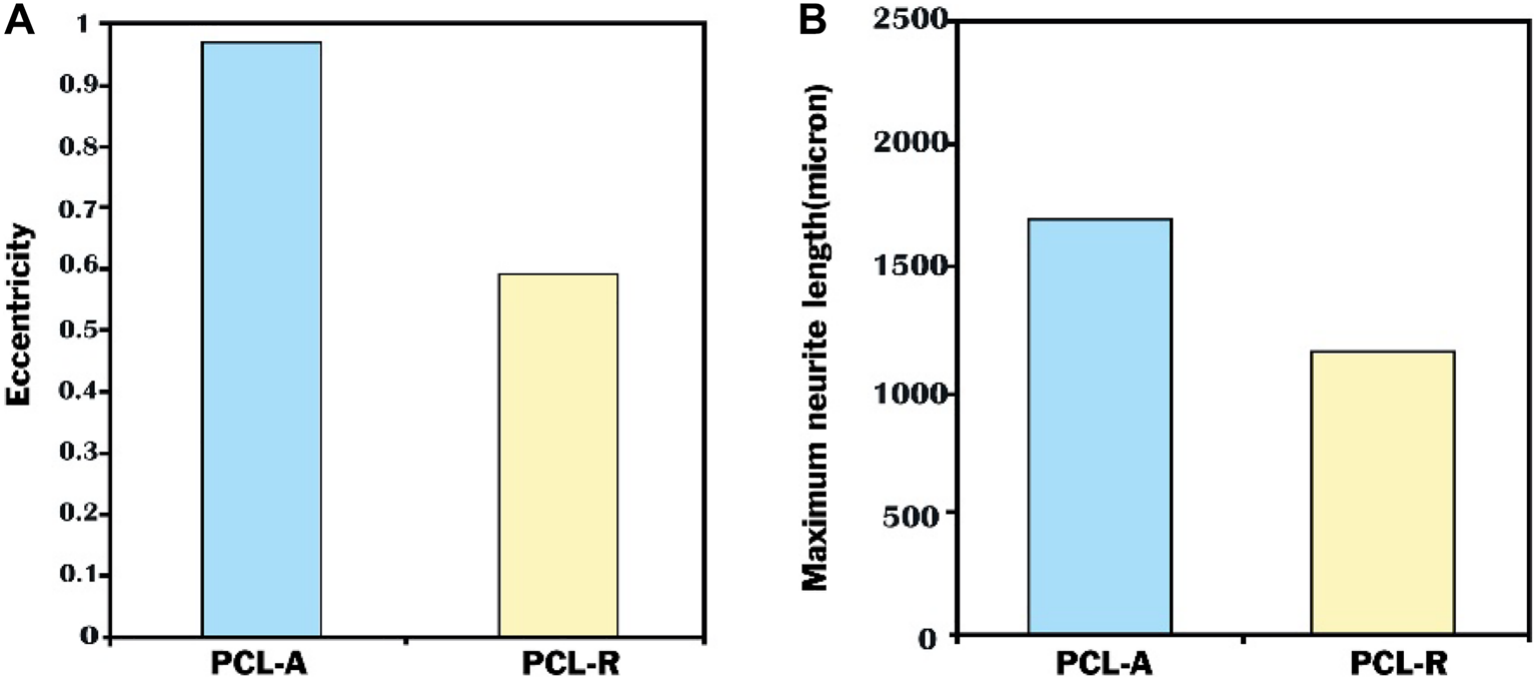
**A** Eccentricity of the neurite field. **B** Maximum neurite length. *Indicates *p* < 0.05 for eccentricity of the neurite field on PCL-R and maximum neurite length from PCL-R as compared to the eccentricity of the neurite field on PCL-A and maximum neurite length from PCL [20]

Nanostructured materials are classified as zero dimensional (0D), one dimensional (1D), two dimensional (2D), or three dimensional (3D) based on the dimensions of their structural elements (Fig. 2) [13].

Nanomaterials are usually synthesized, but there are reports that they exist in nature and are made by plants [14, 15]. Because of their biomimetic qualities and particular biological and mechanical capabilities, nanomaterials have distinct advantages in directing stem cell function and tissue regeneration. Researchers have concentrated on using nanomaterials in the biomedical field because an adequate nano–bio interface can ensure cellular behavior control and, as a result, efficient tissue regeneration. Furthermore, recent breakthroughs in nanomaterial fabrication have increased the awareness of materials science and tissue engineering experts on the potential significance of stem cells in regenerative medicine and advances in stem cell biology have fueled research interests in this sector. Quantum dots, inorganic, and organic nanoparticles, polyplexes, carbon nanotubes, and liposomes are the most often utilized nanoparticles in stem cell research. In addition, nanoparticles made from synthetic materials like poly lactic-co-glycolic acid (PLGA) and poly-3-caprolactone (PCL), as well as natural materials like collagen and chitosan, can be utilized in medicinal applications. These nanoparticles could be used in stem cell research in the following ways: genes, proteins, intracellular delivery of DNA, peptides, RNA interference molecules, and micro medicines for survival or differentiation of stem cells and biosensing of the physiological stem cell status [11, 16, 17].

**Fig. 3:**
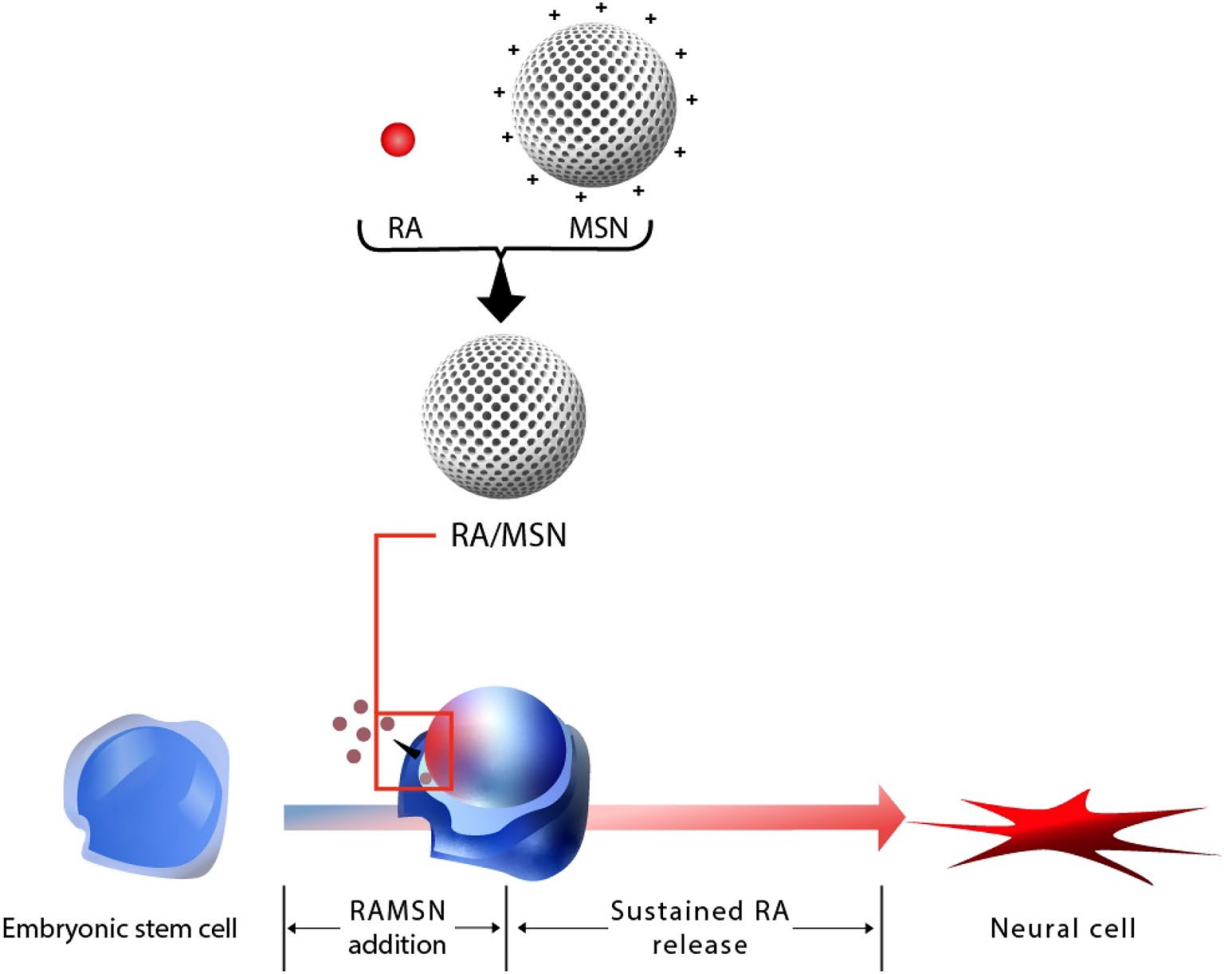
Schematic strategy for neural differentiation of mouse embryonic stem cell (mESC) using mesoporous silica [35]

While there have been previous reviews addressing the influence of nanomaterials on stem cell differentiation, our study stands out in several respects. Firstly, our review exclusively focuses on embryonic stem cells and their neural differentiation in the presence of nanomaterials, a niche that has not been extensively covered. This narrower focus has allowed for a more detailed and comprehensive understanding of the mechanisms and implications involved. Secondly, our analysis offers a classification based on the dimensionality of nanomaterials (0D, 1D, 2D, and 3D), providing a structured framework that facilitates a clearer comparison and understanding of their respective impacts. Lastly, by integrating the most recent studies up to 2023, our review captures the latest advancements and insights in the field, ensuring that readers are equipped with the most current understanding of the topic (Fig. 3).

## Materials and methods

This systematic review was conducted based on the PRISMA (Preferred Reported Items for Systematic Review and Meta-analysis) guidelines.

### Search strategy and selection

**Fig. 4:**
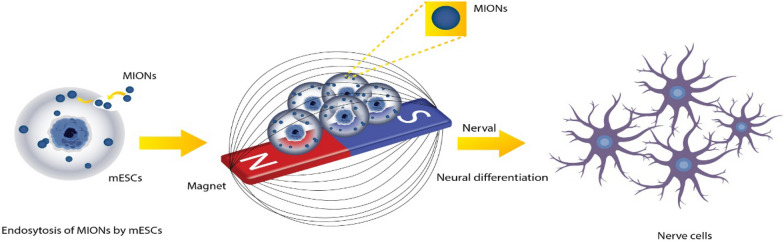
Schematic illustration shows the neural differentiation of mESCs induced by uptake of MIONs, following treatment with an external magnetic field. The combination of magnetic iron oxide nanoparticles and a magnetic field could efficiently promote the differentiation of embryonic stem cells into nerve cells [39]

This systematic review was performed to determine qualified studies on the effect of nanomaterials on neuronal differentiation of embryonic stem cells. Using the formulas presented in Table 1, all available studies were searched in PubMed (Medline), Scopus, Embase (Elsevier), and Web of Science databases. Researchers searched these databases by hand through the reference lists and gray literature. These search engines were searched without language limitations from January 1990 to August 2022. The search protocol was developed based on the four primary roots of “Nanomaterials," “Embryonic," “Neural Differentiation” and “Stem Cell.” All related components were added to search queries based on scientific MeSH terms, EMTREE, or the keywords. The results were limited to human subjects. Reference Manager Bibliographic software was used to manage searched citations. Duplicate entries were searched by considering the title of the published papers, authors, the year of publication, and specifications of the source types. We reviewed the primary search results, and after reviewing each article by its title and available abstract, some of them were eliminated. The evaluation of the papers under consideration was separately performed based on the inclusion and exclusion criteria by the two researchers (RRD, YM) (Fig. 4).

**Table 1:**
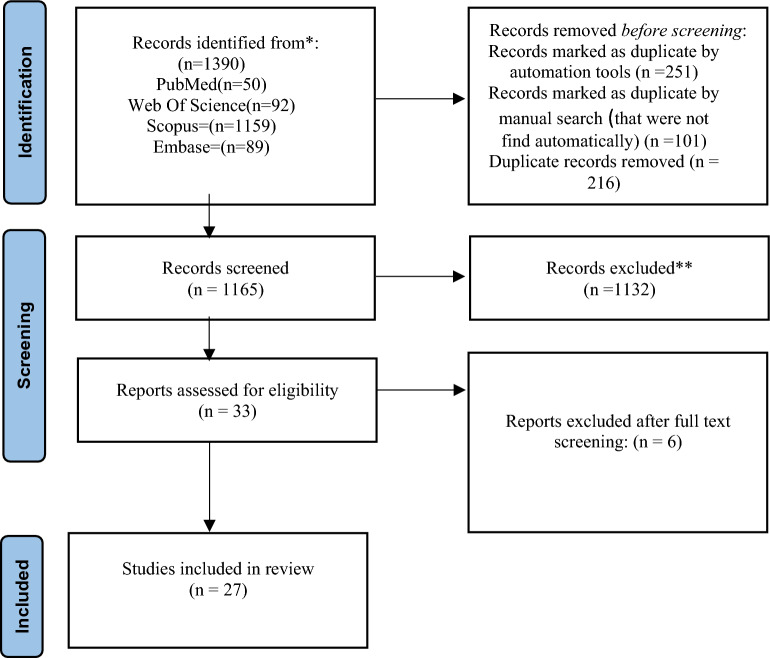
Identification of studies via databases and registers

### Inclusion and exclusion criteria

We included all studies assessing the effect of nanomaterials on embryonic stem cell neural differentiation. We excluded duplicate citations, non-peer-reviewed articles whose abstract and full text was unavailable, and other primary outcomes.

### Data collection and extraction

The found articles through the search were entered into the EndNote software, and duplicates were eliminated. Then, two independent reviewers performed the first stage of screening according to their titles and abstracts. The full texts of selected articles were reviewed to be evaluated based on the inclusion and exclusion criteria. In the case of disagreement, a third researcher’s ideas were considered for selecting studies. For each qualified article, the researchers collected quantitative and descriptive data.

In particular, the researchers extracted data including (1) authors and the publication year; (2) the source of cells; (3) the nanostructure; (4) biomaterial (biomaterial types); (5) the application model; (6) impacts on differentiation) the effect mechanism to differentiate ESCs); (7) differentiation review techniques (the characterization method to evaluate cell differentiation); (8) neuronal expression marker check; and (9) resulting neuron type (the type of differentiated neuron cells). The study research methodology is illustrated in Table2.

**Table 2:** Effect of nanomaterials on embryonic stem cell neural differentiation

Resulted neuron type	Neuronal expression marker check	Differentiation review technique	Impact on differentiations	Biomaterial				Nanostructure	Source of cells	Year of publication	Authors’ name	References
				Composite	Polymers	Ceramic	Metal					
Not mentioned	Β3-Tubulin	ICC^a^	Indirect	PAA-g-CNT^d^	–	–	–	2D nanofilm	hESC	2009	C. Wang, et al	[18]
		CLSM^b^										
		SEM^c^										
Not mentioned	Nestin	ICC	Direct	Collagen/CNT	–	–	–	1D nanofiber	hESC	2009	R. Wang, et al	[19]
		AFM^e^										
		PCM^f^										
Neural precursors	Β3-Tubulin	ICC	Indirect	–	PCL	–	–	1D nanofiber	mESC^h^	2009	S. E. Sakiyama-Elbert, et al	[20]
early neurons	Nestin											
oligodendrocytes	GFAP	ICC										
astrocytes	O4											
Motor neurons	Synapsin I	ICC	Direct	PMAA-g-CNT^i^	–	–	–	1D nanofiber	hESC	2010	T. I. Chao, et al	[21]
	Β3-Tubulin	CLSM										
	Oct4↓	SEM										
Neural progenitors mature neurons	Β3-Tubulin	ICC PCM RT-PCR^j^	Indirect	PLLA^k^-g-heparin	–	–	–	1D nanofiber	hESC	2010	H. J. Lam	[22]
	Nestin											
	GFAP											
	NES											
	SOX2											
	TUBB3											
	NEF3											
	Tyrosine-hydroxylase											
	Oct4↓											
	Nanog↓											
Mature neurons	Β3-Tubulin	ICC SEM CLSM RT-PCR	Direct	–_	Polyurethane acrylate	–	–	3D nanoscale ridge/groove pattern arrays	hESC	2010	M. R. Lee, K. W. Kwon	[23]
	Tuj1											
	Nestin											
	MAP2											
	HuC/D											
	NeuroD1											
	Oct4↓											
	Pdx1 ↓											
	Brachyury ↓											
	GATA6↓											
	DCN↓											
Not mentioned	Β3-Tubulin	ICC SEM CLSM	Indirect	Silk–CNT	–	–	–	3D composite scaffolds	hESC	2012	C. S. Chen	[24]
	Nestin											
Oligodendrocytes mature neurons	Β3-Tubulin	ICC ICC ICC	Indirect	Activated charcoal/collagen/chitosan	_	Activated charcoal	–	3D composite scaffolds	hESC	2012	E. Y. T. Chen	[25]
	Tuj1											
	peripherin											
	Nestin											
	MAP2											
	MBP	SEM										
	Olig-2											
	Neurofilament											
	Pax6											
	FM1-43											
Not mentioned	MAP2	ICC SEM RT-PCR	Indirect	PLLA-g-CNT	–	–	–	1D nanofiber	mESC	2012	M. Kabiri	[26]
	Nestin											
	Β3-Tubulin											
	NSE											
Mature neurons	Β3-Tubulin	ICC SEM	Indirect	PLLA-g-YIGSR peptide	–	–	–	1D nanofiber	mESC	2013	L. A. Smith Callahan	[27]
	Pax6											
	Nestin											
	TUJ1											
	MAP2											
	Oct4↓											
Mature neurons	Β3-Tubulin	ICC FCM SEM RT-PCR	Indirect	MWCNT–PE^l^	–	–	–	3D composite scaffolds	mESC	2013	R. Zang	[28]
	Tuj1											
	Nurr1											
	Nestin											
	Oct4↓											
Motor neurons	Hb9	ICC SEM RT-PCR	Indirect	–	–	Mesoporous silica	–	0D nanoparticles	mESC	2014	A. E. Garcia-Bennett	[29]
	Islet1											
	Islet2											
	Lhx1											
	Lhx3											
	Hoxc5/8											
	ChAT											
Dopamine neurons	TH MAP2	ICC	Indirect	–	–	Graphene oxide	–	0D nanoparticles	mESC	2014	D. H. Yang	[30]
		SEM										
		RT-PCR										
Motor and sensory neurons	MAP2	ICC FCM SEM	Indirect	XCA/mag^m^	XCA	–	–	3D composite scaffolds	mESC	2015	T. Glaser	[31]
	Tuj1											
	Islet1											
	Pax6											
Reduction of viability and neural differentiation	Β3-Tubulin	ICC PCM	Negative effect	–	–	–	Fe_2_O_3_	0D nanoparticles	mESC	2015	A. A. Rostami	[32]
Mature neurons oligodendrocytes astrocytes	Islet1 Islet2 Lim1 Lim2 Nkx6 Pax6 olig2 Gfap Nse Omg MAP2 Nestin↓	ICC SEM RT-PCR	Indirect	–	PCL	–	–	1D nanofiber	mESC	2016	N. Abbasi	[33]
Reduction of viability and neural differentiation	MAP2 AFP NESTIN NCAM BRACHYURY PITX2 LEFTY NODAL	ICC PCM RT-PCR	Negative effect	–	–	–	AuNP^n^ (1.5nm)	0D nanoparticles	hESC	2016	M. C. Senut	[34]
Not mentioned	Β3-Tubulin Tuj1 Oct4↓	ICC SEM RT-PCR	Indirect	–		Mesoporous silica	–	0D nanoparticles	mESC	2017	S. J. Park	[35]
Neural progenitors mature neurons Astrocytes oligodendrocyte	Β3-Tubulin Tuj1 GFAP O4 NESTIN Pax6 Oct4↓ Nanog↓	ICC CLSM SEM RT-PCR FCM	Indirect	–	PLGA	–	–	1D nanofiber	mESC	2017	L. E. Sperling	[36]
Dopaminergic neurons	MAP-2 TH Nurr1	ICC TEM RT-PCR FCM	Indirect	–	–	–	AuNP (30nm)	0D nanoparticles	mESC^o^	2017	M. Wei	[37]
Neural progenitors mature neurons Astrocytes Oligodendrocytes dopaminergic neurons	Sox1 Pax6 Tubb3 Gap43 GFAP MAP-2 Th OLIG1 Cdh2 Syp Pou5f1↓ SSEA-1↓	ICC RT-PCR	Indirect	GYIGSR peptide-g-PCL	–	–	–	1D nanofiber	mESC	2018	E. A. Silantyeva	[38]
Not mentioned	Β3-Tubulin Tuj1 Oct4↓	ICC RT-PCR	Direct	–	–	–	MION^p^	0D nanoparticles	mESC	2019	R. Dai	[39]
Not mentioned	Β3-Tubulin	ICC RT-PCR	Indirect	GNP–pMAG/pSS	–	–	–	3D composite scaffolds	mESC	2019	S. Zhang	[40]
Photoreceptor precursor cells	CRX Ki-67↓ Oct4↓	ICC RT-PCR SEM	Indirect		–	CNF^q^/CNT	–	1D Nanofiber Nanotube	hESC	2020	Y. Chemla	[41]
Not mentioned	Β3-Tubulin NESTIN Flk1 Sox17 Oct4↓	ICC RT-PCR	Direct	AuNP–PMS/FGF2^r^	–	–	–	3D composite scaffolds	mESC	2020	F. Yu	[42]
Mature neurons Astrocytes Oligodendrocytes	Β3-Tubulin NESTIN MAP-2 MBP GFAP	ICC RT-PCR	Indirect	GNP–pMAG/pSS	–	–	–	3D composite scaffolds	mESC	2020	S. Zhang	[43]
Significant retardation in differentiation	Sox2 Oct4 Nanog Gata6 Sox17 Mesp1 Brachyury T Fgf5 Krt14	ICC TEM RT-PCR FCM	Negative effect	–	–	Graphene	–	0D Quantum Dots	mESC	2021	T. Ku	[44]
Dopaminergic neurons	Th	ICC	Indirect	–	TDNs^s^	–	–	3D composite	mESC	2021	M. Wei	[45]
	Nurr1											
	Pitx3	RT-PCR										
Not mentioned	Β3-Tubulin	ICC	Indirect	–	–	–	MNPs^t^	0D nanoparticles	mESC	2022	A. T. Semeano	[46]
	NESTIN	RT-PCR										
Motor neurons	Olig2	ICC	Indirect	–	–	Layered double hydroxide	–	0D nanoparticles	mESC	2023	Y. Bai	[47]
	Nkx6.1											
	Isl1											
	Β3-Tubulin	RT-PCR										
	HB9	FCM										

^a^Immunocytochemistry

^b^Confocal microscope

^c^Scanning electron microscope

^d^Poly (acrylic acid) grafted carbon nanotubes

^e^Atomic force microscopy

^f^Phase-contrast microscopy

^g^Flow cytometry

^h^Mouse embryonic stem cells

^i^Poly (methacrylic acid)-grafted CNT

^j^Real-time polymerase chain reaction

^k^Poly (l-lactic acid)

^l^Multiwalled carbon nanotube-coated polyethylene

^m^Xanthan/magnetite nanoparticles

^n^Gold nanoparticles

^o^The TH promoter-engineered GFP reporter ESCs were constructed by introducing a TH promoter-derived GFP gene into mESCs

^p^Magnetic iron oxide nanoparticles

^q^Carbon nanofibers

^r^Gold nanoparticles (AuNPs), poly(2-methacrylamido glucopyranose-co-3-sulfopropyl acrylate) (PMS), and basic fibroblast growth factor (FGF2)

^s^Tetrahedral DNA nanostructures

^t^Magnetic nanoparticles

### Quality of studies

Three authors evaluated the studies and qualitatively reported their findings based on the Strengthening the Reporting of Observational Studies in Epidemiology (STROBE) Statement.

### Data analysis

We used the content analysis method to analyze the data qualitatively. Content analysis is an objective and rule-guided method used to make replicable and valid inferences and analyze the characteristics of visual, verbal, and written documents.

## Results

The initial search identified 1507 references in PubMed (105 refs), Scopus (1201 refs), Embase (92 refs), and Web of Science (99 refs). 251 duplicate references were automatically found, and 101 ones were manually found. Two hundred and sixteen duplicates were excluded. 1165 studies were evaluated based on their titles and abstracts, and 38 were selected for reading their full texts. After a full-text review, 29 articles were identified as eligible for data extraction and analysis based on the systematic review. The data of every selected study are shown in Table 1. Articles written in languages other than English were excluded. Nine studies used 0D nanomaterials, ten used 1D nanomaterials, two reported 2D nanomaterials, and eight demonstrated the application of 3D nanomaterials. The main biomaterial in studies was polymer-based composites. Three studies reported the adverse effects of nanomaterials on neural differentiation. Studies showed that most nanomaterials could greatly help embryonic stem cell neural differentiation directly and indirectly. Study methods were most economical and could be repeated even in laboratories of developing countries. The result of this review can help scientists all over the world develop their regenerative medicine centers and eventually help patients.

## Discussion

The stochastic differentiation of ESCs has frequently threatened their therapeutic characteristics, limiting the alternatives for nerve tissue engineering [48]. Different approaches have been developed to produce neuronal differentiation from ESCs. One of the most popular techniques is the − 4/ + 4 retinoic acid (RA) approach entailing the development of an embryoid body (EB) for 4 days to activate cells in a state of differentiation, followed by an additional 4 days of RA treatment to induce neural growth [49–51]. ESCs have the capacity to spontaneously give rise to cells from the ectoderm, mesoderm, and endoderm lineages. The microarchitecture of a cell matrix is crucial in regulating the overall development and differentiation pattern of hESCs when biofactors in the media are constant. There is an increasing realization that, in addition to biochemical agents, physical cues provided by scaffolds can direct stem cell differentiation [19, 26]. Cell signaling is the critical molecular pathway that controls cell differentiation and fate determination.

Furthermore, stem cells are regulated by their microenvironment, referred to as a niche. In the niche of stem cells, a combination of physical and chemical signals impact and guide them to retain or select their fate. The balance between external environmental signals and internal cellular components is critical for cell fate regulation. Internal signals stimulated by ECM–cell interactions, cell–cell interactions, and soluble substances change gene expression and cellular activities [52]. The ECM is a three-dimensional biological scaffold composed of a complex composition of proteins and glycoproteins released by resident cells. The specific compositions-dependent response, which is critical for cell fate determination via cell–matrix interactions, is aided by a heterogeneous structure [53, 54]. Nanomaterials mimic these processes to influence neuronal functions like differentiation, proliferation, and electrical characteristics. Nanomaterials have been found to stimulate the signaling pathways and transcription factors involved in neurodevelopment [55]. The Rho kinase pathway is activated by the 2D surface, which leads to cell cycle entrance and following accumulation. 3D surroundings, on the other hand, stimulate the Rac kinase pathway, promoting activities related to morphogenesis and migration [56]. Some nanoparticles can more easily cross cell membranes, which makes them highly desirable because these substances are biocompatible and mechanically stable to enable stem cell proliferation and differentiation [29]. Endocytosis which can be divided into different categories depending on the type of cells and the biomolecules involved in endocytosis is the primary mechanism by which nanoparticles enter cells (e.g., proteins, lipids, and other molecules) [44]. The ability of nanoparticles to stimulate or inhibit the generation of reactive oxygen species (ROS) significantly affects cells. Various developmental processes, including proliferation and differentiation in several illnesses, such as Parkinson’s, are significantly influenced by changes in ROS [32].

Nanomaterials are mostly used as scaffolding. One of the key requirements for a scaffold is that it must act as an anchor for stem cells to be retained close to the injury site rather than attracting and allowing cells to migrate to healthy regions. One of the key elements in controlling cell adhesion is protein absorption on substrates [18, 57]. Nano-scaffold acts as an anchor for stem cells, preventing them from migrating to healthy areas. Another criterion for the long-term effect of scaffolds on cell development is cell survival [18]. The other issue is the risk of tumorigenicity, particularly the formation of teratomas, following transplantation into patients [58]. The primary technique for overcoming this limitation and improving the clinical application of ESCs is to minimize tumorigenicity by controlling ESC differentiation to a specific cell lineage. As a result, Kumamaru et al. described a successful strategy for differentiating and maintaining ESC-derived spinal cord neural stem cells (NSCs) using WNT and FGF2/8 activation and dual suppression of SMAD signaling pathways or corticospinal regeneration [59].

In most of the studies, they used 1D nanomaterials (mostly nanofibers and nanotubes), and by putting them together, they made 3D nano-scaffolds. 3D nanomaterials as an artificial ECM have a high surface area-to-volume ratio. They provide an environment favorable to cellular processes in vivo, such as cell attachment, protein absorption, and subsequent differentiation, promoting cell–cell interactions.[33] Nanofibers are the most critical nanomaterials in stem cell differentiation. They are primarily polymeric or polymer grafted nanoparticles. The studies have shown that nanofiber orientation plays a critical role in differentiation. Nanofibers could support the neural lineage with two distinct orientations, although aligned PCL scaffolds more successfully encouraged the differentiation of neural precursors into adult MN and interneurons. Moreover, compared to the guidance provided by random nanofibers, it may support more dramatic contact-based nerve elongation [33]. Neurite field eccentricity, a reliable indicator of the behavior of individual neurites, can be calculated using the ability of aligned nanofiber surfaces to direct and align populations of expanding neurites. Additionally, neurites projecting from EBs cultured on aligned nanofiber samples had a maximum length of 500 mm, with a significant difference from those projecting from EBs cultured on random nanofibers. Therefore, aligned nanofiber samples may improve the rate and direction of neurite extension. Aligned nanofibers prepared by electrospinning could enhance the differentiation into neural lineages and direct neurite outgrowth [20]. L. E. Sperling et al. also reported creating PLGA electrospun fiber mats with two fiber topographies: randomized and aligned. These fibers demonstrated biocompatibility and exhibited minimal cytotoxicity when utilized as an extracellular matrix replacement for mESCs’ neural development. This study highlights the importance of electrospun fiber alignment in regulating cellular activity and mESC commitment to the neurogenic lineage [36]. In therapies for spinal cord injuries, aligned nanofiber substrates may prevent ESCs from differentiating into astrocytes, limiting the potential for glial scar development. Similar results were found with self-assembling laminin-derived peptide nanofibers, showing that the substrates might similarly reduce astrocytic differentiation [20, 60].

Many studies have suggested that transplantation of ESCs could also be used to treat peripheral nerve and spinal cord injuries. The study by Hayley J. Lam et al. showed that neurite extension could be directed and guided across considerable distances by aligned nanofibers. Therefore, a combination of ES cell therapy and nerve conduits made of aligned nanofibers may offer a more effective method for repairing peripheral nerves than simply injecting cells directly into the injury site, because the scaffolds can offer a more favorable environment for ES cell survival and trophic support. In addition to explaining how bFGF and EGF affect axon formation and neural differentiation, this study also showed how to immobilize active bFGF and EGF onto aligned nanofibers to support neural tissue regeneration [22].

Unidirectional patterns on surfaces cause stem cells to differentiate into a neural lineage. Recent studies have shown that the dimension of nanostructures also affects the development of neural stem cells [20, 61–63]. Therefore, topological dimensions and alignments of these structures may be able to accurately regulate how stem cells differentiate into neurons. M. R. Lee et al. created nanoscale ridge/groove pattern arrays with precisely regulated dimensions and alignments using a UV-assisted capillary force lithography technique. Variation in the dimensions and alignment angles of these ridge/groove patterns was almost negligible. Their approach appears superior to aligned nanofiber approaches in controlling the dimension and alignment of nanoscale patterns [23].

CNTs are one of the popular nanomaterials in the differentiation of stem cells. A structureless and soft gelatin matrix results in the hESC differentiation to cells in all three lineages. In contrast, collagen or a collagen/CNT matrix characterized by one-dimensional fibril structures causes preferential development of hESCs to elongated cells with long filaments. It has been established that carboxylic acid (–COOH) groups are unfavorable cues for neuron differentiation. In agreement with this, T. I. Chao et al. found that the least amount of neuron differentiation and the least amount of cell attachment were produced by PAA surfaces. Instead, they discovered that thin film scaffolds made of the same substance and attached to CNTs exhibited a considerable improvement in neuron differentiation, even outperforming frequently used PLO substrates. The CNT-created nanoscale fiber shape can improve protein adsorption and cell adhesion according to surface analysis and cell adhesion research. This may contribute to the ability of PAA-g-CNT surfaces to aid in differentiating neurons from hESCs. This is crucial because the PAA-g-CNT-based scaffold might offer the transplanted stem cells a long-term shelter to live in and differentiate [18]. The reported discovery that high densities of mesenchymal stem cells increase dopaminergic neuron development supports this assertion [64]. Neuron development has been aided by electric stimulation, resulting in aligned neuron growth to bridge the damaged location rather than transplanted cells sprouting randomly and haphazardly. The conductivity of CNTs does not quickly decrease in severe settings like that of other materials such as conductive polymers, so nanocomposites, including PMAA-g-CNT, with the help of an electric field, can cause a direct differentiation [21]. The neuronal differentiation of mESC is supported and improved when the conductivity of CNT and the alignment of PLLA nanofibers are combined. In particular, differentiated mESC showed increased expression of mature neuronal markers, including Map-2 and NSE, even without direct electrical stimulation when grown on CNT/PLLA conductive composite scaffolds [26]. Directly isolating neuron cells from hESC monolayers, as opposed to using EB culture, which frequently results in heterogeneous differentiation, will result in cells with higher purity and less chance of creating cells at various developmental stages.

Furthermore, hESCs are considered to be “softer” and hence more vulnerable to external signals and more easily coerced into specific lineages than EBs, which are cells already in the differentiating process and covered in layers of ECM molecules [21, 65]. Johnen et al. [21] showed in a crucial work that the CNT surface impacted the gene profile and cell survival of retinal progenitors, suggesting the potential use of these surfaces for covering retinal implant electrodes. According to additional research by Y. Chemla et al. [41], these materials can be used to modify electrode surfaces or work as scaffolds for retinal stem cell implantation.

An attractive biomaterial for neuronal differentiation of hESCs is activated charcoal (AC). Using AC–ECM as a potential bio substrate or scaffold for hESC neuronal development was reported first by E. Y. T. Chen et al. Glial-supported hESCs showed superior neural development in one of the three AC–ECM matrices and AC collagen substrate studied. Their approach, specifically designed to differentiate neurons, avoids cytotoxicity and has the potential to be extended into 3D AC–collagen structures to improve cellular functionalization. This work shows the proof-of-concept application of AC material as a biomatrix for encouraging neuronal development from hESCs only in an in vitro experimental stage. In contrast to CNTs and graphene, AC is an ingestible detoxifying agent that has previously received clinical approval for use. Examining in vivo biocompatibility, biodegradability, tissue deposition, cellular infiltration, and functional host integration will necessitate lengthy animal investigations. Critically needed also is more evidence of the medical applications of AC–ECM substrates. As a result, AC might offer valuable advantages in scaffolds or transplanting devices for medicinal purposes. Their research presents an in vivo feasible natural carbon-based AC composite biomaterial which may support and considerably increase neural development, pointing to potential tissue engineering applications [25]. On the contrary, graphene quantum dots (GQD), another carbon-based nanomaterial, may have retarded development by interfering with the differentiation program of mESCs. It is essential to pay more attention to the adverse health effects of exposure to this nanomaterial during pregnancy or the early stages of development [44].

Some of these nanoparticles are used as carriers in neural differentiation. Mesoporous silica nanoparticles loaded with RA and PUR acted as effective in vitro delivery systems to mediate the differentiation of mouse ESCs into MN precursors. This improves the prospects for their use in in vivo transplantation settings for inducing differentiation of undifferentiated stem cells at the time of transplantation [29]. S. J. Park et al. also simplified the process and accelerated neural induction using mesoporous silica. Their redesigned cell conversion approach only required one RA/MSN complex treatment, which streamlined the procedure and sped up neural induction so that it could be completed in 6 days with good quality. With the help of their technique, neural cells with consistent expression of neurite marker genes were successfully generated from mESCs [35].

The nanoparticle size plays a critical role in their effectiveness. According to the Cao et al.’s study, C17.2 neural stem cells differentiated at 80% when grown with a mean diameter of 300 nm on electrospun nanofibrous PLLA. It was only 40% on micron-sized fibers and had a mean diameter of 1.5 lm [66]. M. C. Senut et al. studied size-dependent toxicity of gold nanoparticles on human embryonic stem cells and their neural derivatives. They observed loss of cohesion, rounding up, and detachment in hESC colonies exposed to 1.5 nm MSA-capped AuNPs, which might indicate ongoing cell death. Within 48 h of treatment, the hESCs exposed to 1.5 nm AuNPs failed to form EBs and quickly fragmented into single cells. Their findings indicated that while MEF feeder cells contained AuNPs, hESCs did not have a substantial amount of AuNPs, indicating that hESC uptake of nanoparticles might differ from that of other cell types. Their findings also indicated that 4 nm AuNPs caused a global decline in DNA methylation, which might help cells by lowering DNA methylation levels.

In conclusion, this study reveals a particular class of AuNPs highly hazardous to hESCs and shows how hESCs can be used to anticipate the neurotoxicity of nanoparticles. This research may eventually affect users of products containing nanoparticles, patients, and employees in the manufacturing industry [34]. However, on the other hand, M. Wei et al.’s study showed that gold nanoparticles could enhance the differentiation of embryonic stem cells into dopaminergic neurons. After 5 days of development by ESCs cocultured with PA6 cells, the effects of AuNPs diameters (5, 15, 30, and 60 nm) on the differentiation of ESCs into dopaminergic neurons (DA) were examined. Comparing the 30-nm-sized AuNPs to the control group, there was a substantial differentiation in boost. Furthermore, in the coculture with PA6 feeder condition, AuNPs did not exhibit any favorable effects on the differentiation of DA neurons in ESCs at 14 days. Previous studies have demonstrated that mouse embryonic fibroblasts are more likely to contain AuNPs than ESCs, leading researchers to hypothesize that the small concentrations of AuNPs in ESCs make it difficult to influence their differentiation. In contrast, a large concentration of AuNPs accumulated in PA6 feeder cells may interfere with the release of differentiation factors, thereby influencing the differentiation of ESCs into DA neurons. All of these findings indicated that during the differentiation of ESCs into DA neurons, adding the proper amount of AuNPs can effectively promote differentiation in feeder-free conditions. Activating the mTOR/p70S6K signaling pathway by AuNPs may result in the upregulation of TH expression, encouraging ESCs to differentiate toward DA neurons [37]. Through the covalent grafting of HS-mimicking polymers and FGF2 to the surface of gold nanoparticles, a novel nanoparticle nanocomposite (AuNP–PMS/FGF2) was proposed and created. The AuNP–PMS/FGF2 nanoparticle composite may significantly accelerate the differentiation of mESCs into nerve cells compared to other bioactive compounds. The AuNP–PMS/FGF2 nanoparticle composite demonstrates good binding ability with cell surface receptors and, consequently, high effectiveness in stimulating neuronal differentiation because AuNPs are involved [42]. S. Zhang et al. have also worked on the pathway by which AuNPs affect. They found that, in accordance with earlier research, GAG mimic-modified GNP could adhere and bind to the receptor on the cell membrane more effectively than the control group. This led to the activation of the downstream signaling pathway. More crucially, when attached to the cell membrane, the gold nanocomposite encouraged RA’s photothermal “conversion” into the cells. This might improve the use of embryonic stem cells in RA molecules, promoting neurogenic differentiation [43].

Magnetic nanoparticles have been regarded as one of the comprehensive biomaterials in biomedicine due to their benign biocompatibility, dimensional controllability, and high stability. They have also demonstrated significant promise in magnetic separation, targeted transportation, bio-imaging, cancer treatment, and regenerative medicine [67–71]. Their sensitivity to magnetic fields also makes it possible to employ them to control the behavior of cells [68] remotely. Lee et al. discovered that, under some circumstances, the application of magnetic tweezer technology might stimulate axon growth, demonstrating the promise of magnetic nanoparticles in repairing nerve injuries [72]. Cho et al. discovered that magnetic nanoparticles treated with polyethylene glycol (PEG) might encourage hMSCs to differentiate into neurons when exposed to electromagnetic fields [73]. The human brain tissue contains magnetite and maghemite nanoparticles by nature. Iron is stored and released by the ferritin protein complex, which plays a role in its creation [74]. Tubulin, a forerunner of microtubules, undergoes structural modifications, which Dadras and colleagues have discovered in the presence of many magnetites [75]. Lower concentrations of magnetite are advantageous for brain cell activity; however, higher amounts can cause cell diseases (death and dysfunction). An electrical field is provided in XCA, a hydrogel with a high negative charge density, to allow for appropriate differentiation. When exposed to electrical stimuli, neurons growing on XCA/mag scaffolds perform better. It has been hypothesized that intrinsic electrons of magnetic particles, which are present in the local magnetic field, aid in ion translocation through the plasma membranes of axonal microtubes [31]. All results show that the combination of magnetic iron oxide nanoparticles and a magnetic field could efficiently promote the differentiation of embryonic stem cells into nerve cells [39]. However, research has revealed that magnetic nanoparticles are quickly endocytosed into cells, and high levels of endocytosis or aggregation will prevent cell differentiation and even strongly induce cell apoptosis [76].

## Conclusion

Neural differentiation is significant in neurological regenerative medicine. The subject of neurological regenerative medicine is predicted to benefit from advances in stem cell research greatly. Clinical trials for various disorders, including age-related macular degeneration (AMD) and spinal cord injury (SCI), have already begun with mixed results. Nanomaterials with different characteristics, particularly those which regulate cellular activities and stem cell fate, have much potential in neural tissue engineering. Understanding the pathophysiological changes in diverse neurological illnesses and designing appropriate nanomaterials for successful modifications in stem cell behaviors could impact neural tissue engineering procedures. These findings indicate a new understanding of potential applications of physicochemical cues in brain tissue engineering.

## Data Availability

The datasets used and analyzed in the current study are available from the corresponding author upon a reasonable request.

## Abbreviations

hESCs: Human embryonic stem cells
iPSCs: Induced pluripotent stem cells
ESCs: Embryonic stem cells
NPCs: Neural progenitor cells
NSCs: Neural stem cells
0D: Zero dimensional
1D: One dimensional
2D: Two dimensional
3D: Three dimensional
PLGA: Poly lactic-co-glycolic acid
PCL: Poly-3-caprolactone
PRISMA: Preferred Reported Items for Systematic Review and Meta-analysis)
XCA/mag: Xanthan/magnetite nanoparticles
MWCNT-PE: Multiwalled carbon nanotube-coated polyethylene
MION: Magnetic iron oxide nanoparticles
AuNP: Gold nanoparticles
mESC: Mouse embryonic stem cells
SMAD: Suppressor of mothers against decapentaplegic
EBs: Embryoid bodies
MION: Magnetic iron oxide nanoparticles
CNF: Carbon nanofibers
FGF2: Fibroblast growth factor
bFGF: Basic fibroblast growth factor
EGF: Epidermal growth factor
TDNs: Tetrahedral DNA nanostructures
RA: Retinoic acid
EB: Embryoid body
MEF: Mouse embryonic fibroblasts
ECM: Extracellular matrix
ROS: Reactive oxygen species
DA: Dopaminergic neurons
SCI: Spinal cord injury
AMD: Age-related macular degeneration
